# Vertebrate Community Responses to Livestock Grazing in an Ancient Mediterranean Rangeland System: Rethinking the Role of Grazing in a Biodiversity Hotspot

**DOI:** 10.3390/biology14081057

**Published:** 2025-08-15

**Authors:** Erin Victor, Scott Brenton, Panayiotis Pafilis, Johannes Foufopoulos

**Affiliations:** 1 School for Environment and Sustainability, University of Michigan, Ann Arbor, MI 48109, USA; 2 Department of Anthropology, University of Maine, Orono, ME 04469, USA; erin.victor@maine.edu; 3 Foxfire Mushrooms, LLC, Longmont, CO 80504, USA; sbrenton@umich.edu; 4 Department of Zoology and Marine Biology, National and Kapodistrian University of Athens, 157 72 Athens, Greece; ppafil@biol.uoa.gr; 5 Museum of Zoology, National and Kapodistrian University of Athens, 157 72 Athens, Greece

**Keywords:** disturbance–diversity relationships, livestock grazing, reptiles, small mammals, passerine birds, Greece, habitat management, vertebrate diversity, intermediate disturbance hypothesis

## Abstract

Mediterranean ecosystems have been grazed by livestock for thousands of years. While considered both a major anthropogenic stressor and a potential habitat conservation tool, the effects of livestock grazing on vertebrate populations remain poorly understood. Our study focused on goat and sheep grazing on a large island off the coast of Greece in order to shed light on (1) the nature of the relationship between livestock grazing and vertebrate assemblages, and (2) the mediating mechanisms. Sampling small mammal, reptile, and passerine bird species across a range of livestock grazing intensities in a Mediterranean pastoral system, we used Generalized Linear Modeling to test for the presence of a unimodal relationship between grazing disturbance and vertebrate diversity in line with the Intermediate Disturbance Hypothesis (IDH). An information-theoretic approach helped elucidate which habitat characteristics best predicted vertebrate-grazing responses. Terrestrial species abundance decreased steadily with increasing grazing, while species richness exhibited a unimodal response, peaking at intermediate livestock stocking rates and offering support for the IDH. This response was best predicted by invertebrate food availability. Both passerine bird species’ richness and abundance showed no clear relationship with grazing yet were significantly correlated with changes in vegetation structure. Our findings suggest that there is no ideal grazing level for broadly optimizing both vertebrate abundance and richness. However, only light-to-intermediate livestock stocking rates are associated with healthy wildlife populations while also promoting terrestrial species richness. Agricultural policy that avoids overgrazing while encouraging a mosaic of different grazing intensities at the regional level is needed to best support diverse vertebrate assemblages.

## Simple Summary

Livestock husbandry is a primary source of livelihood for more than a billion people globally and has been practiced in the Mediterranean since antiquity. Despite this long history, the impact of livestock grazing on wildlife populations and their habitats is poorly understood. The aim of this study was to understand how small mammals, reptiles, and bird species respond to differing degrees of livestock grazing pressure in the Mediterranean, a major biodiversity hotspot. We found that, as livestock grazing pressure increased, the number of small mammals and reptiles decreased. However, the number of these species peaked at moderate grazing levels in alignment with the Intermediate Disturbance Hypothesis. Bird populations, on the other hand, showed no direct response to livestock grazing levels but were influenced by changes in vegetation structure. Our findings suggest that there is no ideal level of livestock grazing that will benefit all wildlife species. Instead, a mosaic of light-to-moderate grazing across the entire region may best support wildlife conservation. This research has important implications for agricultural and environmental policy concerning the role of livestock grazing in wildlife conservation planning.

## 1. Introduction

Livestock husbandry constitutes a globally important livelihood source, with 1.3 billion people relying on livestock as their principal means of subsistence [1]. However, livestock production is often viewed as a significant threat to ecosystem function [2,3]. The growing demand for livestock products and the amount of land designated for animal feed production [4,5] have brought the negative impacts of livestock production into sharp focus. Deforestation, water pollution, erosion, biodiversity loss, and greenhouse gas emissions are all associated with the intensification of domestic grazing systems [3,4,6].

Nevertheless, numerous studies also document the utility of grazing disturbance in promoting biodiversity [7,8]. Livestock grazing can enhance species richness [9], typically at intermediate intensities [10,11]. Commonly referred to as the Intermediate Disturbance Hypothesis (IDH) [12], the observed unimodal relationship between diversity and disturbance is attributed to the role of disturbance in preventing competitive exclusion, allowing for the coexistence of a greater number of species [10].

Although unimodal disturbance–diversity relationships are evident among a variety of ecosystems, taxonomic groups, and disturbance types [13], the IDH has received criticism on empirical and theoretical grounds [14]. Meta-analyses indicate that unimodal disturbance–diversity relationships are rare, with neutral, positive, or negative relationships between disturbance and species richness more commonly observed [15,16]. Variations in methodology may account for the range of disturbance–diversity relationships. For example, the IDH may not be evident if a study only analyzes a narrow range of habitat types or does not consider both disturbance frequency and intensity [17]. However, despite the lack of empirical support, Fox [14] suggests that the theoretical underpinnings of why diversity responds unimodally to disturbance regimes are flawed. He suggests nature’s response to disturbance is more complex than the proposed mechanisms of species coexistence in the presence of disturbance, as outlined under the IDH. Yet, Pierce [18] warns that abandoning the IDH altogether may signal to policymakers that anthropogenic management of natural resources—such as grazing, burning, and mowing—is not important for conservation.

The majority of the grazing disturbance literature concentrates on vegetative responses to livestock grazing [9,19]. Few studies look at how grazing influences animal communities [20], most of which focus on invertebrates [21,22]. How vertebrate assemblages respond to grazing disturbance remains poorly understood. Several studies show grazing negatively impacts bird [23], reptile [24,25], and small mammal [26] abundance and richness, while others find species abundance increases with livestock grazing [27,28]. These studies are typically exclosure studies comparing vertebrate assemblages in grazed and ungrazed plots. Such exclosure studies are unable to detect how assemblages respond to intermediate grazing disturbance levels, nor do they account for the spatial and temporal variations in grazing pressure under natural pastoral systems [7,29]. Additionally, these studies often focus on one particular species or group of animals, e.g., birds or small mammals, which is less informative for conservation managers interested in conserving multiple trophic guilds.

Studies on vertebrate responses to grazing generally fail to investigate the mechanisms driving grazing responses [13]. While the IDH provides a useful starting place for understanding why disturbance can promote species coexistence, a growing number of field studies suggest disturbance is a more complex phenomenon than the IDH acknowledges [14,30]. A more nuanced understanding of the mechanisms driving disturbance–diversity relationships is ultimately needed to inform land management strategies promoting wildlife diversity and ecosystem function.

We focus this study on the Greek island of Naxos, located in the Mediterranean Basin, a major biodiversity hotspot featuring many rare and endemic species [31,32,33]. In this region, livestock grazing has been a linchpin of human society since antiquity, with archeological evidence of domesticated animals dating back to at least 6500 BC [34]. Mediterranean scrublands have adapted to this long history of grazing [35], with many plant species exhibiting physical and chemical defenses (e.g., spiny leaves, thorns, phytotoxins) that make them resistant to herbivory [36,37].

On Naxos, like many other Aegean Sea islands, livestock husbandry of small ruminants (e.g., sheep and goats) provides an important source of subsistence and income from milk, cheese, and meat export [2,34,38]. Mixed flocks of sheep and goats are common throughout the region given their different, and thus complementary, foraging behaviors and plant species preferences, with sheep grazing and goats browsing opportunistically on the native shrubland species [39,40]. However, demographic shifts, global market fluctuations, and agricultural policies have simultaneously driven both the regional intensification and the abandonment of livestock farming practices across the Mediterranean [34,41,42]. Subsidies determined by animal numbers, in particular, have led livestock husbandry practices to shift from predominantly extensive grazing systems, where small, nomadic flocks graze primarily on natural vegetation, to intensive livestock farming systems with larger, more sedentary flocks that require high feed inputs. This has resulted in the abandonment of less-favored areas (LFAs) and heavy overgrazing of more favorable areas (e.g., accessible, fertile lowlands) [34,41]. Abandonment of pastoral lands can lead to woody plant encroachment, reducing biodiversity and increasing wildfire risk [22,42]. Alternatively, intensification of livestock production, where livestock rely primarily on artificial feed, can cause biodiversity losses, desertification, and water pollution [3,4]. Although the Mediterranean landscape has been shaped by anthropogenic stressors over millennia, the vegetation remains dynamic, often responding rapidly to changes in grazing regimes [6,35]. Understanding the responses of livestock-adapted communities to grazing, especially in an island ecosystem, provides an important contrast to other studies investigating these issues in less human-dominated systems and is imperative given the high biodiversity value of the region.

**Figure A1:**
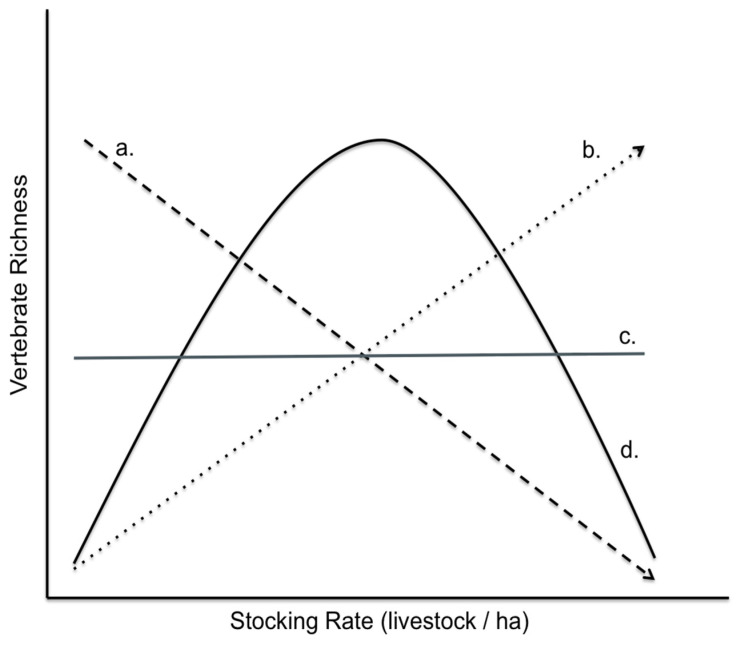
Hypothesized diversity–disturbance relationships for vertebrate species responses to livestock grazing: (a) negative linear, (b) positive linear, (c) neutral, and (d) unimodal.

The objectives of this study were to (1) determine the nature of the relationship between small ruminant livestock grazing and vertebrate assemblages and (2) elucidate the mediating mechanisms. In contrast to previous studies focusing on a single species or animal group, we utilize a multitaxon approach, examining the response of multiple vertebrate groups simultaneously to a broad spectrum of grazing intensities. We hypothesized that vertebrate abundance would decrease with increasing livestock grazing pressure, while vertebrate species richness would peak at intermediate livestock stocking rates (Figure A1), in alignment with the IDH.

To understand the mechanisms underlying the observed disturbance–diversity responses, we investigated a number of a priori hypotheses informed by the disturbance literature. These hypotheses relate to changes in food availability [20,26], vegetation cover [43,44], and vegetation structural heterogeneity [45,46], which in turn drive vertebrate diversity responses. Using an information-theoretic approach and generalized linear modeling, we determined which habitat characteristics, modified through livestock grazing, best predict vertebrate abundance and richness.

## 2. Materials and Methods

### 2.1. Study Area

**Figure 1:**
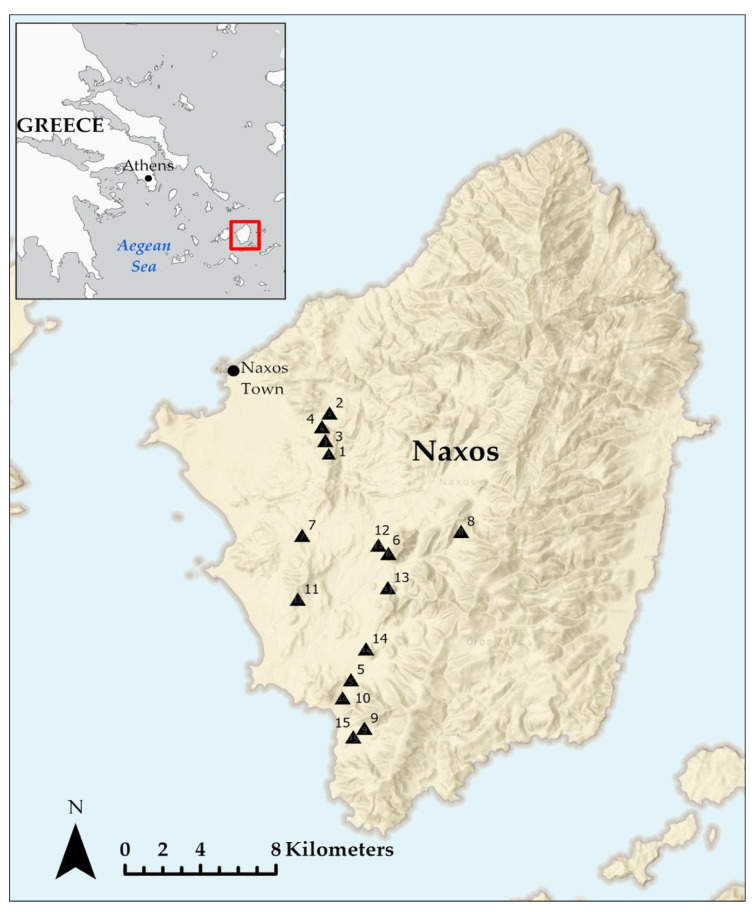
Map of Naxos Island. Study plots are indicated with triangles.

All fieldwork took place during May–July 2013 on the island of Naxos (446 km^2^, 37°08′ N, 25°25′ E), located in the Cyclades archipelago (Aegean Sea, Greece) (Figure 1). A medium-sized island, Naxos was selected because it is representative of the hundreds of islands located in the region and because livestock grazing has been hypothesized to constitute an environmental challenge [38,47]. Climatic conditions are typical of the Mediterranean region, with mild wet winters and warm dry summers [48]. Phrygana, the dominant vegetation type on Naxos and in the Aegean region in general, consists of spiny, summer-deciduous aromatic shrubs and is the product of millennia of livestock grazing [49]. It is locally dominated by *Coridothymus capitatus*, *Genista acanthoclada*, and *Sarcopoterium spinosum*, interspersed with a few evergreen bush species such as *Pistacea lentiscus* and *Juniperus turbinata*. These woody plants are complemented by diverse herbaceous communities across numerous families (e.g., in the genera *Crespis* sp., *Centaurea* sp., *Bupleurum* sp., *Allium* sp., *Papaver* sp., *Plantago* sp., *Trifolium* sp., as well as numerous grasses, e.g., *Hordeum murinum*, *Avena sterilis*) [33,50]. This type of scrubland vegetation is widespread throughout the Mediterranean Basin and is home to a large number of endemic taxa [49].

Naxos has a long history of livestock grazing, with remains of domesticated ungulates first documented from the Neolithic [40]. Pastoralists typically maintain mixed flocks of sheep and goats, and although livestock are often kept unfenced, they are grazed on specific land parcels [38]. Flocks of sheep and goats feed exclusively on natural pasture in the spring and summer, with supplemental feed used only during fall and the winter breeding season [51].

### 2.2. Study Plots

**Figure 2:**
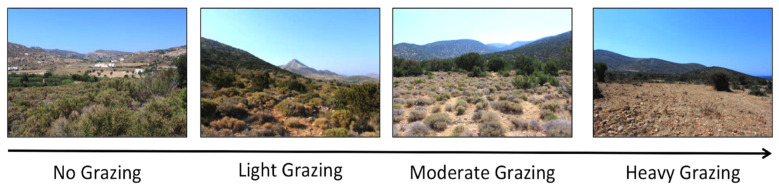
Images of study plots showing different vegetation types along the grazing intensity gradient.

**Figure A2:**
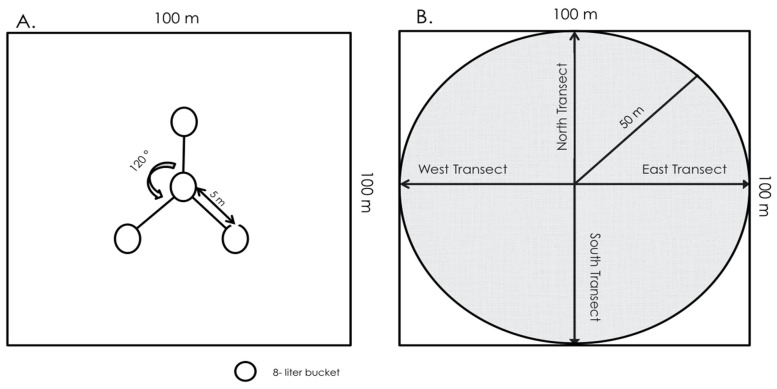
Implementation of vertebrate sampling methods. (**A**) Schematic of drift fence trap array with the locations of pitfall traps. (**B**) Avian point count survey design. Avian point counts were made from the center of the plot, recording birds detected within a 50 m radius (indicated in gray) during a ten-minute observation period.

Fifteen plots were established in low elevation regions of Naxos (Figure 1) to represent a continuum of livestock grazing intensities (Figure 2). Plots were controlled for vegetation type (phrygana), substrate (limestone), and elevation (30–350 m), thus minimizing variation that may confound analyses (Table A1). Plots were 100 × 100 m (1 ha) in size such that they were large enough to reliably sample resident vertebrate taxa while still being homogeneous with regard to grazing pressure, vegetation cover, and species composition. Within each plot, four 50 m transects were established in the four cardinal directions from the plot center to quantify habitat and invertebrate diversity (Figure A2).

### 2.3. Quantification of Grazing Conditions

We used stocking rate as a measurement of livestock (goat and sheep) grazing pressure on each study plot. Stocking rates, defined as the number of goats and sheep stocked per hectare, were based on interviews with pastoralists and validated through our own flock counts. Modifications were made to account for overinflation due to livestock passing through the plot without grazing (Appendix A). Stocking rates were further validated by quantifying livestock dung mass and general reconnaissance [52], which accounted for ground cover, vegetation height, and defoliation patterns in the area.

### 2.4. Vertebrate Population Measures

#### 2.4.1. Terrestrial Vertebrates

Drift fences were used to survey ground-dwelling reptiles and mammals. Fences were constructed on each plot using a three-fence array design (modified from [53]), with three 5 m × 0.5 m lengths of plastic mesh and four 8 L buckets (Figure A2). Metal stakes were used to secure the fences at 1 m intervals. Buckets were sunk flush to the ground. Moist sponges and small rocks were placed in each bucket to prevent desiccation and overheating of animals. Each array was checked daily for a total of 20 trap nights during June and July. Traps were checked during the early morning hours prior to peak ambient temperatures (between 6:00 and 10:00 h UTC) to minimize animal stress. Animals were measured, marked using a permanent marker, and released at the point of capture. All animal handling methods were approved by the University of Michigan’s Institutional Animal Care and Use Committee (IACUC) (Protocol No. 00004850).

#### 2.4.2. Birds

We restricted our investigations to passerine bird species as they were the only birds regularly encountered on the study plots. Birds were surveyed twice at each plot in June and July 2013, overlapping with the breeding season, which lasts from late spring to early summer. Using a fixed radius point count method (modified from [54]), a researcher (E.V.) recorded birds detected (visually and aurally) within a 50 m radius from the center of the plot during a ten-minute monitoring period (Figure A2). Birds flying over the site were excluded from our analysis. Surveys were conducted between 6:00 and 10:00 h UTC under similar weather conditions (clear, low winds, and no precipitation).

### 2.5. Habitat Measurements

We collected habitat condition measurements to assess how livestock grazing influences habitat structure and quality. These included (1) vegetation biomass, (2) vegetation height, and (3) shrub cover.

#### 2.5.1. Vegetation Biomass (Kg/m^2^)

Eight, randomly selected, 80 × 80 cm quadrats (two quadrats along each of the four transects per plot) were sampled. All vegetation within a quadrat was clipped to ground level. All living and dead plant matter was collected, dried in the sun until brittle, and weighed (modified from [55]). Aboveground biomass values, expressed as kg/m^2^ were averaged for all quadrats in each plot.

#### 2.5.2. Vegetation Height (cm)

Vegetation height was sampled at 5 m intervals along 50 m transects radiating from the center of the study plot in the four cardinal directions. Vegetation height was later classified into six height classes: No plant, <10 cm, 10–50 cm, 50 cm–1 m, 1–2 m, and >2 m [56]. Height classifications were used to construct a vegetation structure heterogeneity index using the Shannon Index of Diversity [57] at each site, termed Foliage Height Diversity (*FHD*).

#### 2.5.3. Shrub Cover

Shrub cover was calculated using a gap intercept method [58] to determine the amount of bare ground (gaps) greater than one meter between woody shrubs. Percent gap (% Gap) was calculated by dividing the number of recorded gaps by the total number of measurements on a plot. To obtain the % shrub cover, % Gap was subtracted from 100.

### 2.6. Invertebrate Population Measurements

We installed 12 invertebrate pitfall traps at each site along plot transects (three per transect at the 10, 30, and 50 m points). Pitfall traps were constructed using 320 mL plastic cups filled with 55 mL of ethylene glycol (Formula Flu Antifreeze) to preserve invertebrate samples. Cups were sunk flush to the ground under shrubs. Rocks were loosely placed over each cup to protect samples from livestock trampling. Traps were left out for ten consecutive trapping nights for a total of 120 trapping nights at each site. Invertebrates were identified to order, dried for five hours under a heat lamp, and weighed. Invertebrate biomass (g) was averaged per site. Invertebrate abundance and richness were calculated at the order level. Invertebrates were further classified according to the presence or absence of chemical and morphological defenses based on the presence of a heavily chitinized exoskeleton or noxious chemicals. This classification was then used to calculate the % undefended invertebrates at each site as a proxy for invertebrate food availability.

### 2.7. Statistical Analyses

We tested each variable for deviations from normality and used non-parametric statistics when transformations could not correct these violations. Relationships between livestock stocking rate and vegetation and invertebrate characteristics at each study plot were examined using Spearman’s rank correlation. Livestock stocking rate was log-transformed to account for data overdispersion. Tests were two-tailed with a significance level set at *p* ≤ 0.05. Generalized Linear Modeling [59] was used to determine the relationship between livestock grazing pressure and vertebrate assemblages and potential mediating mechanisms based on a priori models informed by the literature (Table 1). Vertebrate abundance, as is often the case with discrete count data, followed a Poisson error distribution, so when modeling abundance, we used Poisson errors and a log link function. Vertebrate richness, which had a normal distribution, was modeled with normal errors and an identity link function. For model selection we used an Akaike Information Criterion (AIC) approach corrected for small sample size [60,61]. The model with the lowest AICc score was considered to be the “best” model of those compared. To compare the various models, we calculated ΔAICc by subtracting the “best” model’s AICc score from the AICc of the model in question. If ΔAICc < 2, a model was considered to be equally well supported by the data. Akaike weights (w_i_) and evidence ratios were used to determine the degree of certainty in model selection. All statistical analyses were performed using the IBM SPSS v. 31 software package (IBM, New York, USA).

#### 2.7.1. Livestock Grazing-Environment Interactions

We used Spearman’s rank correlations to elucidate the relationship between livestock stocking rate and habitat and invertebrate measurements. Habitat measurements included vegetation biomass, foliage height diversity (FHD), plant species richness, and shrub cover (Table A2). Invertebrate measures included total invertebrate biomass, overall invertebrate abundance, order-level richness, and % undefended invertebrates (Table A3). We additionally investigated the correlation between the abundance of each invertebrate order and stocking rate. Livestock stocking rate was log-transformed to account for data overdispersion.

#### 2.7.2. Relationships Between Livestock Grazing and Vertebrate Populations

The influence of grazing on vertebrate abundance and richness was assessed using Generalized Linear Modeling. Abundance was calculated as the total number of individual birds or terrestrial vertebrates found at each study site, respectively. Species richness was the number of unique terrestrial vertebrates or bird species found at each site. In each case, we tested three models: a linear relationship between vertebrate populations and stocking rate, a unimodal relationship, and the intercept-only model. To test for unimodality, we compared a model using a second-order term against a linear model.

#### 2.7.3. Mechanism

After determining the relationship between livestock grazing pressure and vertebrate assemblages, we ran a number of a priori models to explore potential mechanisms that could explain how grazing influences these vertebrate populations (Table 1).

## 3. Results

### 3.1. Effects of Livestock Grazing on Vegetation Traits and Invertebrate Numbers

Livestock grazing pressure correlated with several vegetation and invertebrate measures assessed at each study plot. As livestock stocking rates increased, vegetation biomass, shrub cover, and plant species richness all decreased significantly (see Table 2). No clear relationship with foliage height diversity (FHD) was observed (Table 2). Livestock grazing also had important quantitative and qualitative effects on invertebrates. While overall invertebrate abundance and order richness did not change significantly with stocking rate (Table 2), invertebrate biomass increased with stocking rate, suggesting an increase in average invertebrate body size. More importantly, there was a significant increase in the percentage of chemically and morphologically defended invertebrates as grazing pressure increased (Table 2). This correlation between livestock stocking rates and % undefended invertebrates is probably explained partially by changes in three specific invertebrate orders. Indeed, rising livestock pressure was negatively correlated with the abundance of Blattodea (ρ = −0.615, *p* = 0.015, n = 15, Spearman), an undefended order of invertebrates. Concomitantly, stocking rate was positively associated with numbers of Coleoptera (ρ = 0.685, *p* = 0.005, n = 15, Spearman) and Hemiptera (ρ = 0.550, *p* = 0.033, n = 15, Spearman) populations, which frequently possess morphological and chemical defenses.

### 3.2. Vertebrate Populations Across Stocking Regimes and Proximate Drivers of Terrestrial Vertebrate and Avian Abundance and Species Richness

We recorded a total of 459 vertebrates (reptiles, mammals, and birds), representing 21 species (Table A4). Of these, 54% (n = 250, 9 species) were terrestrial vertebrates (reptiles and mammals) and 46% were passerine birds (n = 209, 12 species). Certain species were found on all sites, including the Aegean wall lizard (*Podarcis erhardii*) and the Sardinian warbler (*Curruca melanocephala*). No site harbored all study species. Furthermore, sites with the richest avian assemblages, such as Site 1, where eight different avian species were observed, had low terrestrial vertebrate richness, with only three species recorded. Several rare terrestrial vertebrate species, for example, *Ablepharus kitaibelii* and *Sorex minutus*, were found only on grazed sites, whereas none of the less common species were found exclusively on ungrazed sites. In contrast, no such pattern was evident in birds, with several uncommon passerine species found exclusively on grazed (e.g., *Emberiza melanocephala*, *Saxicola torquata*) or ungrazed sites (e.g., *Emberiza calandra*, *Linaria cannabina*) (Table A5).

#### 3.2.1. Terrestrial Vertebrates

**Figure 3:**
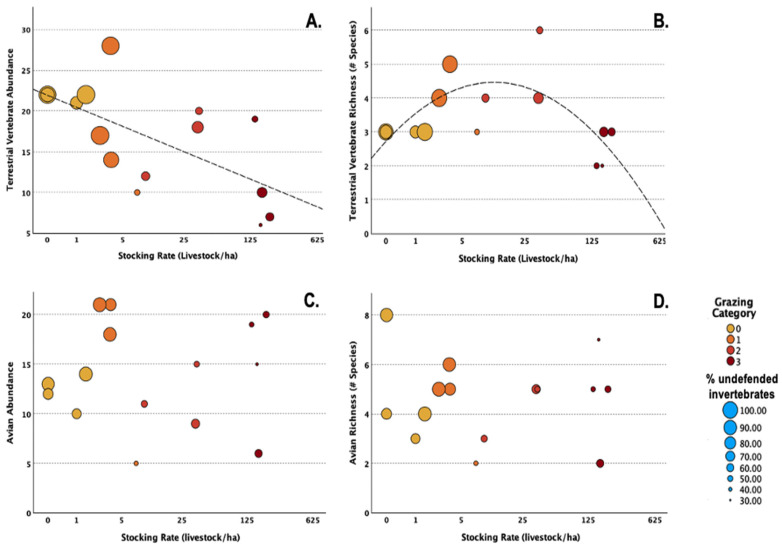
Relationship between livestock stocking rate (livestock/ha) and vertebrate wildlife population characteristics. (**A**) Terrestrial vertebrates: abundance (left) and (**B**) species richness (right). (**C**) Birds: abundance (left) and (**D**) species richness (right). Dashed lines show the best model using an information-theoretic approach to multimodal inference. The absence of a dashed line indicates the null model (intercept-only) provided the best fit (as indicated by the lowest AICc score). The color of each point represents the grazing category: no grazing (yellow); light grazing (orange); moderate grazing (dark orange); and heavy grazing (red). The size of the circle indicates the percent of undefended invertebrates found at each site (30–100%).

Terrestrial vertebrate abundance exhibited a negative linear relationship with livestock stocking rate (Table 3 and Figure 3A). Terrestrial vertebrate abundance was best predicted by invertebrate biomass from pitfalls (Table 4). A unimodal relationship was observed between terrestrial vertebrate species richness and stocking rate (Figure 3B), with the unimodal model providing a better fit for the data than the linear or intercept-only models (Table 3). Vertebrate species richness was greatest at Site No. 11, which had a stocking rate of approximately 36 livestock/ha. However, the fitted curve in Figure 3B suggests that vertebrate species richness actually peaks at much lower stocking rates. Invertebrate biomass was the best predictor of this unimodal relationship between terrestrial vertebrate richness and livestock stocking rate; however, foliage height diversity and the intercept-only models also received considerable support with ΔAIC_c_ < 2 (Table 4).

#### 3.2.2. Avian Species

Avian abundance and richness showed no clear relationship with stocking rate (Figure 3B), with the intercept-only model providing the best model fit in both cases (Table 3). Avian abundance and richness were best predicted by foliage height diversity, with shrub cover also receiving support as an important driver of avian abundance (Table 4).

## 4. Discussion

### 4.1. Response of Vertebrate Assemblages to Grazing

#### 4.1.1. Terrestrial Vertebrates

In this study, we found that while terrestrial vertebrate abundance decreased strongly with livestock stocking rate, species richness peaked at intermediate levels of livestock grazing. In both cases, invertebrate populations appear to be an important mediating mechanism, though more detailed investigations are needed.

Despite increases in reptile abundance among species preferring open habitats [28], most studies show decreased population sizes among reptiles [24,25] and small mammals [26,29] with grazing. We similarly found that the abundance of terrestrial vertebrates (reptiles and small mammals) decreased with increased stocking rates. Although previous studies speculate that food availability may drive the decrease in small mammal and reptile abundance on grazed sites (e.g., [26,62]), this has not been empirically demonstrated. Here, we find that invertebrate biomass was the best predictor of terrestrial vertebrate abundance, which is not surprising given that all the terrestrial vertebrates in this study are, to varying degrees, insectivores (Table A4). Interestingly, invertebrate biomass measured in pitfall traps increases with livestock stocking rate; however, these results likely do not reflect actual arthropod prey availability to wildlife. First, we found that the percentage of morphologically and chemically defended arthropods increases steadily with grazing intensity, suggesting that arthropod biomass alone does not capture the amount of invertebrates truly available for insectivores at a site. In addition, data from Naxos suggest that pitfall traps may provide inflated estimates of arthropod abundance in heavy-grazing contexts [63]. Grazing shifts arthropod communities away from smaller, flying, and non-defended taxa toward larger, armored, desiccation-resistant terrestrial taxa, a group that is preferentially captured in pitfall traps. As such, using different sampling methods (e.g., pan traps, sticky traps) would likely reveal that with intensified livestock grazing, the observed increase in ground arthropods is accompanied by a larger decline in undefended invertebrate numbers [63]. This study suggests terrestrial vertebrates are dependent on the invertebrate populations present. Nonetheless, a finer taxonomic resolution of arthropods, more diversified sampling methods, and a nuanced understanding of vertebrate species’ dietary preferences will help further elucidate the relationship between terrestrial vertebrate abundance and invertebrate populations.

Despite reductions in overall terrestrial population abundance, we found terrestrial vertebrate richness peaked at intermediate stocking rates in accordance with the IDH. This unimodal relationship was best predicted by invertebrate biomass, implying the importance of grazing-mediated changes in invertebrate populations. However, how the increase in invertebrate biomass drives this unimodal response in terrestrial vertebrate richness is unclear. We hypothesize that (1) invertebrate biomass is one of multiple mechanisms driving terrestrial species richness [30] or that (2) invertebrate biomass is, in part, reflecting inverse changes in invertebrate richness.

Our GLM analysis indicates foliage height diversity (FHD), a vegetation structural heterogeneity measure, is also an important predictor of terrestrial vertebrate richness, giving some support to our first hypothesis. Sites with variation in vegetation structure presumably provide greater foraging and nesting opportunities, therefore supporting more diverse terrestrial vertebrate assemblages [44]. As for the second hypothesis, although we found no significant relationship between stocking rate and invertebrate richness, previous studies show increases in invertebrate richness at intermediate grazing disturbance levels [64]. A finer taxonomic resolution [65] and a variety of invertebrate sampling methods may reveal a significant unimodal relationship between grazing and invertebrate species richness in this system. An unanswered question is as follows: Is it the quantity of food available and changes in vegetation structure driving terrestrial species richness responses? Or, is invertebrate biomass acting as a surrogate for the heterogeneity of the food source available under different grazing regimes?

#### 4.1.2. Avian Species

Avian species richness and population abundance, contrary to our expectations, did not show any significant relationship with stocking rate. We hypothesized that avian species richness, similar to terrestrial species richness, would peak at intermediate grazing intensities, mediated through changes in vegetation structure [46]. Additionally, we expected an overall decrease in total avian populations with increased grazing, as seen in previous avian studies [23]. However, we found that neither abundance nor richness was correlated with livestock stocking rates. Instead, both avian abundance and richness were best predicted by vegetation structure complexity. Interestingly, the changes in vegetation structural heterogeneity were not related to livestock stocking rate and were likely the result of management history. The lack of a significant relationship between avian assemblages and livestock grazing is presumably because birds, by virtue of their greater mobility, select and use the landscape on a coarser scale than terrestrial vertebrates.

Although birds have shown both positive (e.g., [46]) and negative (e.g., [23]) responses to livestock grazing, several studies suggest that livestock grazing does not affect bird communities. Reynolds and Trost [66] found similar avian richness on grazed and ungrazed sagebrush sites in Idaho and attributed this finding to the mobility of birds. Similarly, in a pastureland in Australia, avian assemblages were best predicted by tree presence [28]. Since grazing intensification did not impact tree presence, birds showed no response to changes in sheep and cattle grazing pressure [28]. The bird species observed in this study tend to prefer open agricultural habitats (Table A4). Therefore, these species are most likely already adapted to a grazed landscape, and small-scale changes in livestock intensification are unlikely to impact these avian species. While outside of the scope of this study, a more robust sampling of bird populations may be able to reveal differential species responses to grazing presence and should be considered for future studies. Utilizing a naturally occurring livestock grazing continuum, our study scale (1 ha plots) was chosen to reflect current grazing practices in the region. Comparing larger study plots with more homogenous grazing pressure may result in an observable relationship between grazing intensity and avian populations. The current grazing regime in the Mediterranean does not appear to influence passerine richness or abundance, presumably because grazing does not significantly alter the food or habitat availability for avian species in the system at a regional level.

This study focused on vertebrate responses to current small ruminant livestock grazing practices on a large Mediterranean island ecosystem. However, disturbance is a multifaceted phenomenon characterized by its intensity, frequency, extent, duration, and timing [67]. Although we detected no relationship between current stocking rates (disturbance intensity) and avian richness and abundance, other aspects of grazing disturbance, such as frequency or timing, may be correlated with avian assemblages. For example, historically, Naxian shepherds rotated grazing sites, avoiding grazing the same parcels in successive seasons to allow vegetation time to recover. However, with changing animal husbandry practices on the island, many sites are now continuously grazed. Livestock grazing can alter vegetation structure, creating heterogeneous vegetation structures [68], which our study shows is important in determining bird species richness and abundance. Therefore, we assume vegetation structural changes are correlated with the timing, duration, and/or frequency of livestock grazing rather than grazing intensity alone.

Future studies looking at multiple scales and disturbance characteristics are necessary to better understand if and how avian populations respond to livestock grazing regimes. Meanwhile, our study adds to the growing body of literature indicating that vertical vegetation structural heterogeneity plays a critical role in supporting diverse avian populations [44,45], suggesting rangeland managers and conservationists should strive to create and maintain vertical structural diversity, especially in areas where avian populations are threatened.

## 5. Conclusions

In this study, we found that while insectivorous terrestrial vertebrates with more limited dispersal abilities showed evidence of a unimodal disturbance–diversity response, avian richness did not show an apparent disturbance–diversity response. The mechanisms believed to drive unimodal disturbance responses (e.g., resource partitioning, competition–colonization trade-offs, temporal and spatial relative nonlinearity, and storage effects) [30,67], all focus on competitive interactions. While moderate grazing disturbance may benefit vertebrate assemblages, finding evidence of unimodal diversity–disturbance relationships among terrestrial vertebrates depends upon the methods of analysis, such as how species are grouped. Unimodal responses may only be evident when grouping species with similar dietary or habitat preferences, which are therefore in competition with one another. For example, if disturbance enables coexistence through suppression of competitive dominants, the reduction in Sardinian warbler (*Curruca melanocephala*) numbers, a primarily insectivorous bird, is unlikely to benefit the predominantly seed-eating goldfinch (*Carduelis carduelis*).

In demonstrating distinct disturbance–diversity relationships for terrestrial and avian vertebrate groups, as well as unique mechanisms mediating these disturbance responses, our findings support the need for more nuanced disturbance models than provided by the IDH. This is in line with Rivera-Núñez and colleagues’ argument that the IDH lacks “clarity in defining the intensity, duration, and spatial scale of disturbances” [17]. Our results are useful in informing models that are better able to predict the impact of various disturbance regimes. Future studies should investigate a wider range of potential mechanisms, as well as how different grazing regime characteristics such as livestock type(s) (e.g., sheep, goat, pig, cow, bison, etc.) [3,41], frequency, duration, and timing of livestock grazing [38,67], or the concurrence of multiple disturbances [19] influence vertebrate populations.

### Conservation Implications

These results underscore the importance of investigating the effects of disturbance across multiple groups of species. Grazing, particularly by feral goats, has long been recognized as a threat to the conservation of endemic plants and animals on islands [69,70]. Here, focusing solely on avian population responses might suggest that livestock grazing is irrelevant for wildlife conservation. However, by examining both terrestrial and avian vertebrate assemblages simultaneously, this study not only highlights their divergent responses to livestock grazing but also identifies two different mediating habitat characteristics (food availability and vegetation structural heterogeneity) that conservation managers should consider. In addition, we highlight the utility of limited livestock grazing as an important conservation tool in Mediterranean scrublands. That said, caution is warranted when applying these findings to other insular communities. Naxos is a large island with a long history of livestock grazing; the authors expect that vertebrate responses to livestock grazing practices will depend both on island size and grazing history. In general, species communities on smaller and drier islands are less likely to tolerate intense livestock grazing, and stocking rates need to be correspondingly adjusted.

The differential disturbance responses of terrestrial and avian vertebrate species suggest no single livestock stocking rate will maximize all vertebrate groups. Our results indicate that while small mammal and reptile populations decline with increasing livestock herbivory, their species richness peaks at intermediate stocking rates (perhaps as high as 30–40 animals/ha). At the same time, avian assemblages do not appear to show a significant response to livestock grazing in this system. Taken together, this evidence suggests that while overstocking islands can profoundly impact terrestrial wildlife populations, light-to-moderate grazing is not antithetical to, and may even promote, vertebrate conservation efforts. Nonetheless, these results need to be balanced with parallel considerations of how grazing impacts plant diversity and ecosystem functioning.

Overall, livestock grazing’s long history in the region supports the sustainability of this practice. This finding dovetails with previous claims that grazing can be an important conservation practice. Other scholars have suggested that policy should encourage extensification of livestock practices (e.g., lower stocking rates where livestock feed primarily on natural vegetation) rather than intensification (e.g., higher stocking rates supported by artificial feed) and abandonment of pastoral lands [7,51]. A recent study focused on Mediterranean reptile species found that, while climate was the primary factor limiting species distributions, land abandonment demonstrated a strong homogenizing effect on reptile assemblages [71]. While this suggests low-intensity grazing is critical for preserving reptile habitat, we believe a mosaic of grazing intensities would best support maximum vertebrate species richness, considering certain less common species were found exclusively on ungrazed or heavily grazed sites. Such a grazing mosaic has been suggested for optimizing invertebrate [72] and bird richness [64]. A shifting mosaic provides habitat for multiple species, as well as conditions necessary for vertebrates relying on several habitat characteristics throughout different lifecycle stages. Although our study did not investigate the effects of fire disturbance on vertebrate populations, previous work has shown the importance of fire–grazing interactions [8], and conservation managers should consider the combination of prescribed burns and livestock grazing as a way of fostering heterogeneous habitats.

## Figures and Tables

**Table 1:** A priori models corresponding to our hypotheses of potential mechanisms driving the grazing–vertebrate relationship.

Mechanism	Model	Reference
Food Availability	Vertebrate Richness/Abundance~Vegetation Biomass	[26]
Food Availability	Vertebrate Richness/Abundance~Invertebrate Biomass	[20]
Shrub Cover	Vertebrate Richness/Abundance~Shrub Cover	[43,44]
Vegetation Structural Heterogeneity	Vertebrate Richness/Abundance~FHD	[45,46]

**Table 2:** Results of Spearman’s rank correlations (ρ) analyzing the relationship between stocking rate and vegetation and invertebrate measurements at each site. N = 15. Significant results (*p* ≤ 0.05) are indicated with an asterisk.

Response Variable	Spearman’s Rank Coefficient (ρ)	Significance (*p*-Value)
**Vegetation measures:**		
Vegetation biomass	−0.699	0.004 *
Shrub cover	−0.790	<0.001 *
Foliage height diversity (FHD)	−0.51	0.857
Plant species richness	−0.890	<0.001 *
**Invertebrate measures:**		
Invertebrate biomass	0.629	0.016 *
Invertebrate richness	0.325	0.237
Invertebrate abundance	0.396	0.144
% undefended invertebrates	−0.711	0.003 *

**Table 3:** GLM comparisons for the response of terrestrial vertebrates (small mammal and reptile) (A) and avian (B) abundance (1) and richness (2) to changes in livestock stocking rates. Models are ranked in ascending order by ∆AIC_c_. The log likelihood, deviance (D), AIC_c_ score, AIC_c_ weights (w_i_), and evidence ratios are given for each model. Model variables include linear and squared terms for livestock stocking rate. TVA designates terrestrial vertebrate abundance, TVR terrestrial vertebrate richness, AA avian abundance, and AR—avian richness.

	Log Likelihood	D	AIC_c_	∆AIC_c_	w_i_	Evidence Ratio
A1. Terrestrial Vertebrate Abundance						
**TVA~stocking_rate**	**−45.880**	**23.227**	**96.759**	**0.000**	**0.810**	**1.000**
TVA~stocking_rate + stocking_rate^2^	−45.749	22.966	99.680	2.921	0.188	4.308
TVA~intercept	−53.017	37.502	108.342	11.583	0.002	327.504
A2. Terrestrial Vertebrate Richness						
**TVR~stocking_rate + stocking_rate^2^**	**−18.028**	**9.717**	**48.055**	**0.000**	**0.647**	**1.000**
TVR~intercept	−22.540	17.733	50.079	2.024	0.235	2.751
TVR~stocking_rate	−21.635	15.719	51.452	3.397	0.118	5.466
B1. Avian Vertebrate Abundance						
**AA~intercept**	**−47.570**	**29.035**	**97.448**	**0.000**	**0.725**	**1.000**
AA~stocking_rate	−47.536	28.967	100.073	2.625	0.195	3.715
AA~stocking_rate + stocking_rate^2^	−46.835	27.564	101.853	4.405	0.080	9.048
B2. Avian Vertebrate Richness						
**AR ~intercept**	**−28.565**	**39.600**	**62.130**	**0.000**	**0.805**	**1.000**
AR~stocking_rate	−28.560	39.572	65.301	3.171	0.165	4.882
AR~stocking_rate + stocking_rate^2^	−28.372	38.594	68.744	6.614	0.030	27.303

**Table 4:** Model selection for predicting terrestrial (A) and avian (B) richness (1) and abundance (2) as mediated through grazing-induced changes in habitat characteristics. Models are ranked in ascending order by ∆AIC_c_. The log likelihood, deviance (D), AIC_c_ score, AIC_c_ weights (w_i_), and evidence ratios are given for each model. Model variables include invertebrate biomass, foliage height diversity (FHD), vegetation biomass, and shrub cover. TVA designates terrestrial vertebrate abundance, TVR terrestrial vertebrate richness, AA avian abundance, and AR—avian richness.

	Log Likelihood	D	AIC_c_	∆AIC_c_	w_i_	Evidence Ratio
A1. Terrestrial Vertebrate Abundance						
**TVA~invertebrate biomass**	**−37.376**	**10.378**	**79.843**	**0.000**	**1.000**	**1.000**
TVA~shrub cover	−48.291	28.049	101.581	21.738	0.000	52,522.661
TVA~vegetation biomass	−50.797	33.063	106.595	26.752	0.000	644,351.717
TVA~intercept	−53.017	37.502	108.342	28.499	0.000	154,3402.573
TVA~FHD	−52.495	36.459	109.991	30.148	0.000	3,520,100.154
A2. Terrestrial Vertebrate Richness						
**TVR~invertebrate biomass**	**−20.616**	**15.585**	**49.631**	**0.000**	**0.387**	**1.000**
TVR~intercept	−22.540	17.733	50.079	0.448	0.309	1.251
TVR~FHD	−21.576	15.586	51.325	1.694	0.166	2.333
TVR~vegetation biomass	−22.385	17.371	52.951	3.320	0.073	5.259
TVR~shrub cover	−22.504	17.649	53.189	3.558	0.065	5.924
B1. Avian Abundance						
**AA~FHD**	**−45.384**	**24.662**	**95.768**	**0.000**	**0.430**	**1.000**
AA~shrub cover	−46.190	26.274	97.380	1.612	0.192	2.239
AA~intercept	−47.570	29.035	97.448	1.680	0.186	2.316
AA~invertebrate biomass	−46.485	26.865	97.971	2.203	0.143	3.009
AA~vegetation biomass	−47.562	29.018	100.124	4.356	0.049	8.829
B2. Avian Richness						
**AR~FHD**	**−22.880**	**18.556**	**53.941**	**0.000**	**0.971**	**1.000**
AR~intercept	−28.565	39.600	62.130	8.189	0.016	60.009
AR~invertebrate biomass	−28.274	38.095	64.730	10.789	0.004	220.192
AR~vegetation biomass	−28.276	38.103	64.734	10.793	0.004	220.633
AR~shrub cover	−28.300	38.228	64.783	10.842	0.004	226.105

## Data Availability

The original contributions presented in this study are included in the article and the Appendix A. Further inquiries can be directed to the corresponding author.

## Appendix A

**Table A1:** Site characteristics. Location, elevation, aspect, vegetation characteristics, stocking rate, and dung biomass for each study plot.

Site No.	Latitude	Longitude	Elevation (m)	Aspect	Primary Vegetation	Secondary Vegetation	Stocking Rate*	Livestock Dung Biomass (g/m^2^)
1	N37°04.463′	E025°25.348′	179.53	N	*Genista*/*Calicotome*/*Cistus* Phrygana	Kermes Oak	0	0.00
2	N37°04.758′	E025°25.378′	66.49	E	*Cistus*/*Calicotome* Phrygana	Pistacea	0	0.00
3	N37°04.682′	E025°25.256′	150.87	S	*Cistus*/*Calicotome* Phrygana	Kermes Oak	1	0.33
4	N37°05.023′	E025°25.161′	54.86	W	*Genista* Phrygana	Pistacea	1.5	0.00
5	N36°58.819′	E025°25.999’	32.87	S	*Coridothymus* Phrygana	Juniper	2.5	3.11
6	N37°02.026’	E025°26.963’	197.39	W	*Coridothymus*/*Genista* Phrygana	Kermes Oak/Juniper	3.5	1.33
7	N37°02.360′	E025°24.593′	170.38	E	*Cistus*/*Calicotome* Phrygana	Pistacea	3.57	1.00
8	N37°02.457′	E025°29.137′	326.25	E	*Coridothymus*/*Genista* Phrygana	Kermes Oak	7.5	2.94
9	N36°57.625′	E025°26.374′	54.86	S	*Coridothymus* Phrygana	Juniper	9.38	5.83
10	N36.58.375′	E025.25.751′	55.42	W	*Coridothymus* Phrygana	Juniper	35	6.22
11	N37°00.794′	E025°24.470′	140.20	E	*Coridothymus*/*Genista* Phrygana	Juniper	36.1	2.39
12	N37°02.067′	E025°26.875′	206.65	W	*Coridothymus* Phrygana	Juniper	140	14.67
13	N37°01.079′	E025°27.044′	206.95	N	*Coridothymus*/*Genista* Phrygana	None	160	16.72
14	N36°59.578′	E025°26.421′	124.05	W	*Coridothymus* Phrygana	Juniper	166	59.33
15	N36°57.554′	E025°25.949′	61.63	N	Sparse *Coridothymus*	None	200	39.50

* Note: Stocking rates are defined as the number of goats and sheep per hectare and modified based on interviews with local pastoralists. If livestock merely passed through a plot, the stocking rate was divided by a factor of ten to represent the fraction of time animals spent on the site (estimated at a tenth of a day); if animals were only present on a given site for half of the year, we corrected by dividing by a factor of two.

**Table A2:** Vegetation response variables. Vegetation biomass (kg/m^2^), shrub cover (%), Foliage Height Diversity (FHD), and plant species richness at each study plot.

Site No.	Vegetation Biomass (kg/m^2^)	Shrub Cover (%)	FHD	Plant Species Richness
1	4.85	100	1.16	14
2	4.42	100	0.88	13
3	1.96	98	0.81	12
4	2.17	100	0.84	10
5	0.71	71	0.89	8
6	1.53	78	1.27	12
7	4.86	100	1.06	8
8	1.48	88	0.88	10
9	0.77	95	0.59	4
10	0.68	71	0.91	6
11	1.55	68	1.15	8
12	0.61	76	0.73	7
13	0.44	60	1.16	5
14	1.53	76	0.76	4
15	0.60	12	1.07	2

**Table A3:** Invertebrate response variables. Invertebrate biomass (g), invertebrate abundance, invertebrate order richness, and percentage undefended invertebrates at each study plot.

Site No.	Invertebrate Biomass (g)	Invertebrate Abundance	Invertebrate Richness	Undefended Invertebrates (%)
1	0.42	292	7	91.13
2	0.46	265	9	78.57
3	0.36	169	6	73.26
4	0.17	291	6	94.88
5	0.44	395	10	94.49
6	0.42	344	9	92.77
7	1.3	667	10	84.6
8	3.27	735	10	46.4
9	0.42	270	7	57.56
10	0.57	212	9	69.12
11	0.38	87	5	52.75
12	0.64	387	7	47.95
13	2.26	1376	8	37.17
14	1.29	612	10	63.17
15	1.42	401	8	56.47

**Table A4:** List of species observed during the study and associated foraging guilds.

Latin Name	Common Name	Foraging Guild
*Curruca melanocephala*	Sardinian warbler	Insectivore
*Parus major*	Great tit	Insectivore/Granivore
*Carduelis carduelis*	Goldfinch	Granivore/Omnivore
*Galerida cristata*	Crested lark	Granivore/Omnivore
*Lanius senator*	Woodchat shrike	Insectivore
*Linaria cannabina*	Linnet	Granivore/Insectivore
*Emberiza calandra*	Corn bunting	Granivore/Omnivore
*Passer domesticus*	House sparrow	Granivore
*Emberiza cirlus*	Cirl bunting	Granivore/Omnivore
*Saxicola torquata*	Stonechat	Insectivore
*Chloris chloris*	Greenfinch	Granivore/Omnivore
*Emberiza melanocephala*	Black-headed bunting	Granivore/Omnivore
*Crocidura suaveolens*	Lesser white-toothed shrew	Insectivore
*Sorex minutus*	Eurasian pygmy shrew	Insectivore
*Lacerta trilineata*	Balkan green lizard	Insectivore
*Eryx jaculus turcicus*	Javelin sand boa	Carnivore/Insectivore
*Podarcis erhardii*	Aegean wall lizard	Insectivore
*Mediodactylus kotschyi*	Kotschy’s gecko	Insectivore
*Hemidactylus turcicus*	Mediterranean house gecko	Insectivore
*Ablepharus kitaibelii*	European snake-eyed skink	Insectivore
*Vipera ammodytes meridionalis*	Long-nosed viper	Carnivore/Insectivore

**Table A5:** Terrestrial vertebrate and avian species richness (R), abundance (A), and species assemblages by site. Species presence is indicated by an x. Sites are ordered based on livestock stocking rate (StR) from low to high. Terrestrial species abbreviations: Pe—*Podarcis erhardii*; Mk—*Mediodactylus kotschyi*; Cs—*Crocidura suaveolens*; Ak—*Ablepharus kitaibeli*i; Lt—*Lacerta trilineata*; Ejt—*Eryx jaculus turcicus*; Vam—*Vipera ammodytes meridionalis*; Sm—*Sorex minutus*; Ht—*Hemidactylus turcicus*. Avian species abbreviations: Cm—*Curruca melanocephala*; Gc—*Galerida cristata*; Cch—*Chloris chloris*; Pd—*Passer domesticus*; Cca—*Carduelis carduelis*; Em—*Emberiza melanocephala*; Eci—*Emberiza cirlus*; St –*Saxicola torquata*; Eca—*Emberiza calandra*; Ls—*Lanius senato*r; Lc—*Linaria cannabina*; Pm—*Parus major*.

		Terrestrial Species											Avian Species													
Site No.	StR	R	A	***Pe***	***Mk***	***Cs***	***Ak***	***Lt***	***Ejt***	***Vam***	***Sm***	***Ht***	R	A	***Cm***	***Gc***	***Cch***	***Pd***	***Cca***	***Em***	***Eci***	***St***	***Eca***	***Ls***	***Lc***	***Pm***
1	0	3	22	x	x				x				8	13	x	x	x	x	x		x		x		x	
2	0	3	22	x		x		x					4	12	x			x	x				x			
3	1	3	21	x	x			x					3	10	x		x	x								
4	1.5	3	22	x	x				x				4	14	x	x	x	x								
5	2.5	4	17	x	x			x	x				5	21	x	x	x	x		x						
6	3.5	5	28	x	x	x	x				x		6	18	x	x	x	x		x	x					
7	3.57	5	14	x	x	x		x		x			5	21	x	x	x	x	x							
8	7.5	3	10	x	x	x							2	5	x							x				
9	9.38	4	12	x	x	x	x						3	11	x	x	x									
10	35	4	18	x	x	x				x			5	9	x	x			x	x						x
11	36.1	6	20	x	x	x			x		x	x	5	15	x	x	x		x	x						
12	140	2	19	x	x								5	19	x	x	x	x		x						
13	160	2	6	x	x								7	15	x	x	x	x	x		x	x				
14	166	3	10	x	x		x						2	6	x	x										
15	200	3	7	x		x	x						5	20	x	x	x	x						x		
